# NrCAM modulates sonic hedgehog signalling by controlling smoothened translocation in the cilium

**DOI:** 10.1186/2046-2530-4-S1-P35

**Published:** 2015-07-13

**Authors:** B Basu, O Weeranantanapan, D Xenaki, A Furley

**Affiliations:** 1 Biomedical Science, University of Sheffield, Sheffield, UK

## Objective

Cerebellar development involves a spurt of proliferation in external granule layer (EGL) in response to shh, causing granule neuron precursors (GNPs) to proliferate. These cells subsequently differentiate into granule neurons in the inner granule layer (IGL). F3, a CNTN family molecule, can interact with NrCAM to switch GNPs from proliferation to differentiation. We aim to identify the role of NrCAM in the sonic hedgehog response in GNPs.

## Methods

GNPs were extracted from wildtype and NrCAM mutant P5 cerebella using Percoll gradient centrifugation. Proliferation response to shh was measured using EdU in presence/absence of F3-Fc. GNPs treated with shh/SAG were stained with antibodies against Arl13b and smo to look for differences in cilia size and smo occupancy after different treatment times.

## Results

NrCAM^-/- ^and wildtype GNPs both proliferated equally in response to shh. F3 was found to block the proliferation response in wildtype but not in NrCAM^-/- ^GNPs. F3 also failed to affect proliferation in SmoA1 GNPs with a constitutively active smo suggesting that the F3-NrCAM mediated block lay upstream of Smo. NrCAM was detected in wildtype cilia and Smo localization was affected in NrCAM^-/- ^GNPs. No differences in cilia length were observed.

## Conclusion

Our results suggest that NrCAM affects shh-mediated proliferation by controlling smo movement into the cilium.

